# Iron status and anemia control are related to peritoneal membrane properties in peritoneally dialyzed patients

**DOI:** 10.3389/fmed.2023.1148094

**Published:** 2023-07-06

**Authors:** Tomasz Głogowski, Ewa Wojtaszek, Jolanta Malyszko

**Affiliations:** Department of Nephrology, Dialysis and Internal Medicine, Medical University of Warsaw, Warsaw, Masovian, Poland

**Keywords:** end-stage renal disease, peritoneal dialysis, anemia, iron homeostasis, hepcidin, erythroferrone, GDF15, zonulin

## Abstract

**Background:**

Characteristics of peritoneal membrane is unique and individually different in peritoneal dialysis patients. Relationship between specific nature of peritoneal transport, anemia and inflammation has not yet been extensively studied. We attempted to outline the complex interplay of several biomarkers of iron status and their association with peritoneal transport, degree of inflammation and residual renal function.

**Methods:**

A total of 58 patients treated with peritoneal dialysis either CAPD or APD for at least 3 months were enrolled in this study. Full blood count, traditional markers of iron status (transferrin saturation-TSAT and ferritin), serum erythroferrone-ERFE, soluble transferrin receptor (sTfR), hepcidin, zonulin, growth differentiation factor −15 (GDF15), IL-16, hsCRP and hypoxia-inducible factor—α (HIF-1-α; in serum and dialysate) were measured using commercially available tests. We also performed Peritoneal Equilibrium Test and assessed GFR level.

**Results:**

Hb levels above 10 g/dL was found in 74% of patients. Hb levels positively correlated with residual renal function and nutritional status. Adequate iron status was diagnosed in 69% of subjects, only in 9% of patients, criteria for absolute iron deficiency were met. Serum ERFE correlated inversely with hepcidin levels but was not associated with erythropoietin stimulating agent dose. Peritoneal transport had strong correlation with dialysate sTfR (*p* < 0.05), dialysate hepcidin (*p* < 0.05), dialysate GDF15 (*p* < 0.01) and dialysate zonulin (*p* < 0.001) levels, as well as serum IL6 (*p* = 0.03), serum hs-CRP (*p* = 0.04) and dialysate hs-CRP (*p* = 0.04).

**Conclusion:**

Residual kidney function contributes considerably to better control of anemia. Various degree of inflammation is inherent to PD patients. Additionally, fast-average peritoneal transport is associated with greater degree of inflammation and higher concentration of markers of iron status, GDF15 and zonulin in dialysate. This finding may indicate more effective clearance of higher-range middle molecules in fast-average transporters. The role of ERFE as a marker of erythropoiesis in PD patients requires further investigation.

## Introduction

Anemia affects the majority of patients with end-stage renal disease (ESRD). Several mechanisms have been suggested to contribute to anemia of chronic kidney disease (CKD) with relative erythropoietin deficiency, iron deficiency and maldistribution, shortened erythrocyte lifespan, nutritional deficiencies and chronic inflammation (1, 2).

Up to date, the management of anemia and iron metabolism disturbances and outcomes in predialysis CKD and hemodialysis patients have been extensively studied. There are few studies that have examined characteristics of anemia and iron status in peritoneal dialysis (PD) patients. In recently published large international study it has been shown that more than half of PD patients have a various degree of anemia, and, regardless of iron supplementation, a certain percentage of them has disequilibrium of iron status (3, 4). Abnormal iron status is associated with increased risk of all-cause and cardiovascular mortality (5, 6). Furthermore, it is a factor in terms of both risk and prognosis for PD-related infectious complications (7, 8).

Population of PD patients tends to differ from HD patients in several regards, which may impact iron homeostasis and anemia. They tend to have better preserved residual kidney function and less iron loss as a result of dialysis technique. At the same time chronic, subclinical or overt inflammation at both systemic and intraperitoneal levels commonly affects PD patients. The underlying mechanisms of inflammation in peritoneal dialysis patients are complex and result from direct stimulation of cytokine generation by uremic milieu and impaired renal clearance of inflammatory cytokines, as well as cumulative peritoneal membrane injury and dysfunction stemming from exposure to non-biocompatible dialysis fluids, episodes of PD-associated peritonitis, biofilm formation within catheter lumen and endotoxemia by translocation of macromolecules from the intestine (9–11). Furthermore. intravenous iron administration causes oxidative stress and inflammation and may affect intraperitoneal homeostasis (12, 13). Available data suggest that systemic and local intraperitoneal inflammation reflect distinct processes and sequelae, and in particular the significance and impact of intraperitoneal inflammation on iron homeostasis requires further elucidation.

PD is a continuous technique with effective diffusive and convective transport of small- to middle-molecular solutes. However, data regarding the efficacy of removal of molecules involved in iron homeostasis are scarce. It has been demonstrated that, compared to HD patients, PD patients have significantly lower serum hepcidin levels, and dialysate hepcidin concentrations are higher that of HD ultrafiltrate (14). It suggests that hepcidin may be effectively removed via peritoneal membrane, however peritoneal hepcidin clearance was evaluated only in one small study (15). It should be emphasized that individual properties of peritoneal membrane may also influence efficacy of removal of hepcidin and other solutes.

To the best of our knowledge, the influence of peritoneal membrane transport on iron metabolism in peritoneal patients has not been investigated so far.

### Objectives

The aim of the study was to assess iron status and anemia control in end-stage renal disease patients on peritoneal dialysis depending on the properties of the peritoneal membrane and residual renal function.

## Materials and methods

**Figure 1:**
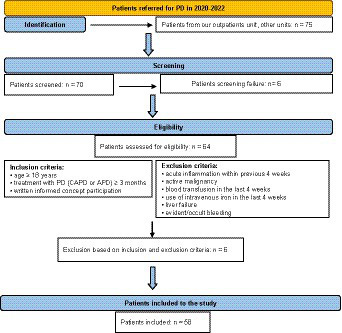
Flow chart on the PD population included in the study.

The patients in our PD program who fulfilled inclusion criteria entered the study. Inclusion criteria were: age ≥ 18 years, treatment with PD (CAPD or APD) ≥ 3 months and written informed consent for participation. Exclusion criteria were: acute inflammation within previous 4 weeks, active malignancy, blood transfusion in the last 4 weeks, use of intravenous iron in the last 4 weeks, liver failure and evident/occult bleeding as shown in flow chart on Figure 1.

The study protocol was approved by the Ethics Committee of Medical University of Warsaw, and carried out in accordance with the Declaration of Helsinki.

### Data collection

Demographics and clinical data including age, gender, primary kidney disease, comorbidity burden assessed by Charlson Comorbidity Index (CCI), and dialysis vintage were collected.

Anemia treatment—oral iron and Erythropoietin Stimulating Agent (ESA) doses were collected. All patients received CERA (Continuous Erythropoetin Receptor Activator—methoxy polyethylene glycol-epoetin beta).

Hemoglobin concentration (Hb), serum ferritin (SF), serum iron, total iron-biding capacity (TIBC) were obtained using standard laboratory methods (automated system) in certified local central laboratory. Transferrin saturation with iron (TSAT) was calculated as the ratio of serum iron and TIBC and expressed as percentage.

According to the definition of absolute and functional iron deficiency patient were categorized into four group based on the levels of TSAT and SF: reference iron status (RIS) defined as TSAT 20%–30% and SF 100–500 ng/mL, absolute iron deficiency (AID)—TSAT < 20% and SF < 100 ng/mL, functional iron deficiency (FID)—SAT < 20% and SF > 100 ng/mL, and high iron status (HIS)—TSAT > 30% and SF > 500 ng/mL.

Commercially available tests were used to measure high sensitivity C-reactive protein—hsCRP (R&D Systems, Inc. Minneapolis, United States), interleukin 6—IL-6 (R&D Systems, Inc. Minneapolis, United States), soluble transferrin receptor—sTfR (R&D Systems, Inc. Minneapolis, USA), hepcidin-25 (Peninsula Laboratories International Inc., United States), erythroferrone—ERFE (SunRed, Shanghai, China), growth differentiation factor 15—GDF15 (R&D Systems, Inc. Minneapolis, United States), zonulin (Immundiagnostik AG, Bernsheim, Germany). The assays were performed in serum and in dialysate.

Standard Peritoneal Equlibration Test (PET), residual renal function (expressed as glomerular filtration rate—GFR) and dialysis adequacy (expressed as Kt/V) were calculated using Patient OnLine software version (Fresenius Medical Care, Bad Homburg, Germany). Serum creatinine, urea, protein and glucose, dialysate creatinine, urea, protein and glucose, and urine creatinine, urea and protein for calculations were obtained using standard laboratory methods (automated system) in certified local central laboratory. Patients were divided into three peritoneal membrane transport groups based on D/P creatinine: slow, ≤0.52; average, 0.53–0.77; fast, ≥0.78. For further analysis, based on the mean ± SD of peritoneal membrane transport in the entire study group, two transport subgroups were distinguished—“fast-average” (D/Pcreatinine ≥ 0.65), and “slow-average” (D/Pcreatinine < 0.65).

### Statistical analysis

Data are presented as mean and standard deviation, median (lower and upper quartile) or frequencies and percentages for categorical variables. Differences between study groups were tested using the Mann–Whitney *U*-test, Kruskal-Wallis test, and differences in the relative frequencies were tested using the Pearson chi-square test. A value of *p* < 0.05 was considered statistically significant.

Analyses of the correlation of each parameter were performed using Person or Spearman correlation coefficients. *p* < 0.05 was considered statistically significant.

All calculations were performed using STATISTICA software package (version 13), StatSoft Poland.

## Results

A total 58 patients met predefined inclusion criteria and were enrolled to the present study.

Demographic and clinical characteristics of the studied group are presented in Table 1.

**Table 1:** Demographic and clinical characteristics of the studied group.

Parameter	Value
Age (years)	49 ± 17
Male sex (*n*, %)	*n* = 28 (48%)
Charlson comorbidity index (CCI)	5 ± 3
**The cause of kidney disease: (*n*, %)**	
Diabetic nephropathy	*n* = 5 (8%)
Glomerulonephritis	*n* = 29 (50%)
Hypertensive/vascular	*n* = 4 (7%)
Interstitial nephropathy	*n* = 10 (17%)
ADPKD	*n* = 5 (8%)
Other/unknown	*n* = 6 (10%)
Dialysis vintage (months)	Median 9 (3–186)
**Dialysis method**	
CAPD (*n*, %)	27 (47%)
APD (*n*, %)	31(53%)
PMT (D/Pcreatinine)	0.65 ± 0.11
GFR (mL/min)	5.84 ± 4.24
Kt/V	2.59 ± 0.84
CERA users (*n*, %)	26 (45%)
Oral iron users (*n*, %)	14 (24%)

CAPD, Continuous Ambulatory Peritoneal Dialysis; APD, Automated Peritoneal Dialysis; PMT, peritoneal membrane transport; GFR, glomerular filtration rate; CERA, Continuous Erythropoetin Receptor Activator; ADPKD, Autosomal Dominant Polycystic Kidney Disease.

### Anemia control

Mean Hb in the studied group was 10.91 ± 1.27 g/dL. The majority of patients (54%) had Hb 10–11.6 g/dL, while 24% had Hb < 10 g/dL, and 22% > 11.6 g/dL. The characteristics of the patients in each of the quartiles of Hb are presented in Table 2.

**Table 2:** Characteristics of patients according to Hb level.

	Hb < 10 g/dL	Hb 10–11.6 g/dL	Hb > 11.6 g/dL	P
Age (years)	46 ± 19	49 ± 16	54 ± 19	NS
CCI	4.5 ± 3	4.5 ± 2.2	5.3 ± 2.9	NS
CERA	62.5 (0–110)	30 (0–75)	0	<0.01
GFR (ml/min)	1.5 (0–4.1)	5.6 (2.8–8.8)	9.4 (6.0–11.6)	<0.001
Kt/V	2.15 ± 0.7	2.64 ± 0.65	2.93 ± 1.19	NS
PMT (D/Pcreatinine)	0.64 ± 0.12	0.64 ± 0.12	0.66 ± 0.1	NS
Serum albumin (g/dL)	3.53 ± 0.6	3.75 ± 0.45	3.99 ± 0.31	<0.05
Serum ferritin (ng/mL)	368 (290–643)	206 (144–337)	173 (111–208)	<0.05
TSAT (%)	36.4 ± 14.5	33.3 ± 12.4	31.0 ± 13.6	NS
Erythroferrone (ng/mL)	1.61 (0.84–3.47)	2.28 (1.47–5.3)	1.34 (0.81–3.17)	NS
Serum sTfR (nmol/L)	46.6 ± 14.2	35.8 ± 8.2	41.8 ± 7.7	NS
Dialysate sTfR (nmol/L)	0.1 (0.06–0.13)	0.06 (0.01–0.18)	0.04 (0.03–0.2)	NS
Serum Hepcidin (ng/mL)	72.2 (59.2–128.1)	94.5 (40.2–140.7)	33.4 (25.9–75.4)	NS
Dialysate Hepcidin (ng/mL)	9.4 (8.8–28.9)	7.9 (5.4–18.5)	10.3 (7.9–38.8)	NS
Serum hs-CRP (ng/mL)	4,998 (2,345–11,200)	1,403 (710–2,972)	1,322 (996–2,858)	NS
Dialysate hs-CRP (ng/mL)	35.6 (10.3–117.9)	11.9 (3.5–78.8)	14.6 (7.9–27.9)	NS
Serum IL6 (pg/mL)	9.14 (8.16–14.84)	5.69 (3.4–11.3)	5.7 (4.1–7.78)	NS
Dialysate IL6 (pg/mL)	85.0 (50.6–102. 9)	37.1 (6.4–75.8)	46.4 (25.8–67.1)	NS
Serum GDF15 (pg/mL)	3,873 (3,630–4,495)	3,182 (2,237–4,193)	2,982 (2,346–3,602)	NS
Dialysate GDF15 (pg/mL)	391 (375.5–454.3)	310.3 (102.8–551.9)	218.8 (201.6–575.0)	NS
Serum Zonulin (ng/mL)	73.3 (64.2–77.2)	58.5 (54.2–64.3)	61.1 (59.4–66.6)	NS
Dialysate Zonulin (ng/mL)	0.75 (0.65–0.97)	0.54 (0.3–0.66)	0.6 (0.54–1.13)	NS

CCI, Charlson Comorbidity Index; GFR, glomerular filtration rate; PMT, peritoneal membrane transport; CERA, Continuous Erythropoetin Receptor Activator; TSAT, transferrin saturation with iron; sTfR, soluble transferrin receptor; hs-CRP, high sensitivity C-reactive protein; IL-6, interleukin 6; GDF15, growth differentiation factor 15.

Patients with mean Hb > 11.6 g/dL had significantly better preserved residual kidney function, higher serum albumin and lower serum ferritin levels. They also tended to have the lowest serum hepcidin, ERFE and GDF15 levels, however differences did not reach statistical significance.

Almost a quarter of the patients in the study group had a hemoglobin concentration < 10 g/dL despite a significantly higher median CERA dose and better iron metabolism control expressed by higher SF and TSAT. Compared to the others, these patients had significantly lower residual renal function and serum albumin levels. They also tended to have more systemic and intraperitoneal inflammation, as suggested by higher serum and dialysate concentrations of IL6 and hs-CRP.

Hb strongly correlated with residual renal function (r = 0.53, *p* < 0.0001), dialysis adequacy (r = 0.4, *p* < 0.01) and serum albumin (0.36, *p* < 0.01). Among iron status biomarkers, statistically significant correlation was revealed only with serum ferritin (SF; r = −0.47, *p* < 0.001). Details are presented in Table 3.

**Table 3:** Correlations between hemoglobin and studied parameters.

	Hemoglobin	*P*
CCI	0.15	NS
CERA	−0.47	<0.0001
GFR (mL/min)	0.53	<0.0001
Kt/V	0.4	<0.01
PMT (D/Pcreatinine)	−0.02	NS
Serum albumin (g/dL)	0.36	<0.01
Serum ferritin (ng/mL)	−0.47	<0.001
TSAT (%)	−0,15	NS
Erythroferrone (ng/mL)	−0.06	NS
Serum sTfR (nmol/L)	−0.09	NS
Dialysate sTfR (nmol/L)	−0.16	NS
Serum Hepcidin (ng/mL)	−0.2	NS
Dialysate Hepcidin (ng/mL)	0,19	NS
Serum hs-CRP (ng/mL)	−0.22	NS
Dialysate hs-CRP (ng/mL)	−0.22	NS
Serum IL6 (pg/mL)	−0.37	NS
Dialysate IL6 (pg/mL)	−0.36	NS
Serum GDF15 (pg/mL)	−0.29	NS
Dialysate GDF15 (pg/mL)	−0.15	NS
Serum Zonulin (ng/mL)	−0.14	NS
Dialysate Zonulin (ng/mL)	−0.35	NS

CCI, Charlson Comorbidity Index; GFR, glomerular filtration rate; PMT, peritoneal membrane transport; CERA, Continuous Erythropoetin Receptor Activator; TSAT, transferrin saturation with iron; sTfR, soluble transferrin receptor; hs-CRP, high sensitivity C-reactive protein; IL-6, interleukin 6; GDF15, growth differentiation factor 15.

GFR strongly correlated with Hb (r = 0.53, *p* < 0.0001) and CERA dose (r = −0.75, *p* < 0.0001), but among iron status biomarkers only with serum ferritin (r = 0.32, *p* < 0.01) and GDF15 (r = −0.53, *p* < 0.01).

### Iron status

Mean TSAT in the entire group was 33.5 ± 13.1%, and mean serum ferritin 345 ± 414.3 ng/mL. The majority of patients (69%) had reference iron status (RIS). Absolute iron deficiency (AID) has been found only in 9% of patients while high iron status (HIS) 14%. None of the patients met the criteria for the diagnosis of a functional iron deficiency (FID). The characteristics of patients stratified by iron status are presented in Table 4.

**Table 4:** Clinical characteristics of patients according to peritoneal membrane transport.

	Fast-average PMT	Slow-average PMT	*P*
Age (years)	53 ± 17	46 ± 17	NS
CCI	5.5 ± 2.7	3.9 ± 2.3	<0.05
CERA ()	0 (0–75)	30 (0–30)	NS
GFR (mL/min)	5.9 ± 4.6	5.7 ± 3.9	NS
Kt/V	2.62 ± 0.9	2.55 ± 0.71	NS
Serum albumin (g/dL)	3.7 ± 0.4	3.79 ± 0.54	NS
Hemoglobin (g/dL)	11.1 ± 1.33	10.8 ± 1.22	NS
Serum ferritin (ng/mL)	186 (125–360)	281 (144–419)	NS
TSAT (%)	32.6 ± 12.8	34.6 ± 13.3	NS

CCI, Charlson Comorbidity Index; GFR, glomerular filtration rate; PMT, peritoneal membrane transport; CERA, Continuous Erythropoetin Receptor Activator; TSAT, transferrin saturation with iron.

Patients in HIS group tended to have worse preserved RRF and lower Hb level compared to others despite higher CERA doses. They had higher serum and dialysate hepcidin and erythroferrone levels, however the differences did not reach statistical significance. In this group, there was also a trend toward more intense inflammation, especially in relation to higher concentrations of IL6 and hs-CRP in the dialysate.

In the entire studied group, ERFE correlated significantly only with hepcidin (0.4, *p* < 0.05), but not other iron status biomarker or CERA dose.

Hepcidin correlated significantly with SF (0.67, *p* < 0.001), TSAT (0.4, *p* < 0.05), sTfR (0.46, *p* < 0.05), hs-CRP (0.51, *p* < 0.05) and CERA dose (0.6, *p* < 0.01). Details are presented in Table 5.

**Table 5:** Correlations between iron status biomarkers and studied parameters.

	Serum ferritin (ng/mL)	TSAT (%)	Serum sTfR (nmol/L)	Serum hepcidin (ng/mL)	Serum GDF15 (pg/mL)	Serum Zonulin (pg/mL)	Erythroferrone (ng/mL)
CCI	−0.21	−0.3	0.34	−0.17	0.18	0.16	−0.2
CERA	0.34	0.02	0.17	**0.6** ^**^	0.42	0.09	−0.06
GFR (mL/min)	**−0.32** ^*^	−0.09	−0.13	−0.13	**−0.54** ^**^	−0.02	0.11
Kt/V	−0.2	0.07	0.03	0.15	**−0.5** ^**^	0.02	0.24
PMT (D/Pcreatinine)	0.01	−0.09	**0.48** ^*^	−0.06	0.14	0.31	−0.18
Serum albumin (g/dL)	0.02	0.08	0.09	0.07	−0.15	0.08	0.06
Serum ferritin (ng/mL)	x	**0.57** ^**^	−0.11	**0.67** ^***^	0.18	0.034	0.34
TSAT (%)	**0.57** ^**^	x	−0.2	0.4	−0.13	−0.2	0.27
Erythroferrone (ng/mL)	0.33	0.23	−0.07	**0.4** ^*^	−0.23	−0.13	X
Serum sTfR (nmol/L)	−0.1	−0.19	X	**0.46** ^*^	0.14	0.32	−0.07
Dialysate sTfR (nmol/L)	−0.2	−0.18	0.37	−0.33	0.18	0.14	−0.12
Serum Hepcidin (ng/mL)	**0.67** ^***^	**0.4** ^*^	**0.46** ^*^	X	0.1	−0.12	**0.4** ^*^
Dialysate Hepcidin (ng/mL)	0.38	0.1	0.09	**0.54** ^**^	**0.51** ^**^	−0.7	0.08
Serum hs-CRP (ng/mL)	0.02	−0.05	0.13	−0.18	−0.12	−0.07	0.07
Dialysate hs-CRP (ng/mL)	−0.03	−0.01	0.28	−0.08	−0.19	−0.1	0.09
Serum IL6 (pg/ml)	−0.12	−0.25	0.34	−0.17	0.12	0.11	0.003
Dialysate IL6 (pg/mL)	**0.46** ^**^	−0.34	0.25	0.09	−0.09	0.06	0.14
Serum GDF15 (pg/mL)	0.17	−0,13	0.14	0.1	X	0.21	−0.23
Dialysate GDF15 (pg/ml)	0.03	−0.12	0.22	−0.09	**0.53** ^**^	0.04	−0.07
Serum Zonulin (ng/mL)	−0.03	−0.2	0.32	−0.12	0.21	X	−0.13
Dialysate Zonulin (ng/mL)	−0.35	−0.07	0.42	−0.11	0.17	0.02	−0.02

^*^p < 0.05, ^**^p < 0.01, ^***^p < 0.001. CCI, Charlson Comorbidity Index; GFR, glomerular filtration rate; PMT, peritoneal membrane transport; CERA, Continuous Erythropoetin Receptor Activator; TSAT, transferrin saturation with iron; sTfR, soluble transferrin receptor; hs-CRP, high sensitivity C-reactive protein; IL-6, interleukin 6; GDF15, growth differentiation factor 15. Bold means statistically significant.

### Iron status and peritoneal membrane transport

The majority of patients (65%) had average peritoneal membrane transport (D/Pcreatinine 0.53–0.77), 21% had slow peritoneal membrane transport (D/Pcreatinine ≤ 0.52), and 14% had fast peritoneal membrane transport (D/Pcreatinine ≥ 0.78). In each of the iron status categories the proportion of patients with particular type of peritoneal membrane transport was similar (RIS: average PMT 67%, slow PMT 18%, fast PMT 15%; HIS average PMT 63%, slow PMT 25%, fast PMT 12%; AID average PMT 60%, slow PMT 20%, fast PMT 20%).

For further analysis, based on the mean ± SD of peritoneal membrane transport in the entire study group, two transport subgroups were distinguished—“fast-average” (D/Pcreatinine ≥ 0.65), and “slow-average” (D/Pcreatinine < 0.65). The clinical characteristics of the subgroups are presented in Table 4, and data on iron biomarkers in serum and dialysate assessed by us are presented in Table 6.

**Table 6:** Biomarkers of iron metabolism and inflammation according to peritoneal membrane transport.

	Fast-average PMT	Slow-average PMT	*P*
Serum sTfR (nmol/L)	43.4 (36.6–44.8)	31.1 (28.9–42.4)	0.05
Dialysate sTfR (nmol/L)	0.15 (0.06–0.2)	0.04 (0.01–0.11)	0.05
Serum Hepcidin (ng/mL)	72.2 (31.6–101.2)	72.06 (35.2–134.4)	NS
Dialysate Hepcidin (ng/mL)	10.72 (8.2–48.3)	5.53 (5.44–10.09)	NS
Serum Erythroferrone (ng/mL)	1.48 (1.07–3.53)	2.3 (1.44–4.65)	NS
Serum hs-CRP (ng/mL)	2,394 (1,403–4,498)	1,358 (672.3–2,972)	0.05
Dialysate hs-CRP (ng/mL)	31.75 (11.26–98.35)	10.25 (2.24–41.84)	0.05
Serum IL6 (pg/mL)	9.16 (6.88–15.75)	4.22 (2.88–6.31)	0.01
Dialysate IL6 (pg/mL)	46.36 (17.3–83.96)	48.24 (13.95–67.61)	NS
Serum GDF15 (pg/mL)	3,602 (2,346–4,214)	2,999 (2,290–4,193)	NS
Dialysate GDF15 (pg/mL)	422.65 (187.9–633.4)	201.6 (95.29–543)	NS
Serum Zonulin (pg/mL)	64.29 (60.4–69.68)	55.47 (54.17–64.31)	0.05
Dialysate Zonulin (pg/mL)	0.78 (0.59–1.54)	0.54 (0.26–0.66)	0.02

PMT, peritoneal membrane transport; sTfR, soluble transferrin receptor; hs-CRP, high sensitivity C-reactive protein; IL-6, interleukin 6; GDF15, growth differentiation factor 15.

“Fast-average” transporters tended to have more severe inflammation and higher serum and dialysate levels of sTfR and zonulin, however, only serum IL6 and dialysate zonulin reached statistical significance.

PMT strongly correlated with CCI (r = 0.34, *p* < 0.01) and serum albumin (r = −0.25, *p* = 0.04) as well as serum IL6 (0.4, *p* = 0.03), serum hs-CRP (0.39, *p* = 0.04) and dialysate hs-CRP (0.38, *p* = 0.04). Strong correlation was also revealed between PMT and dialysate sTfR (0.38, *p* < 0.05), dialysate hepcidin (0.46, *p* < 0.05), dialysate GDF15 (0.5, *p* < 0.01) and dialysate zonulin (0.64, *p* < 0.001) levels.

Moreover, dialysate hepcidin correlated with serum ferritin (0.38, *p* < 0.05), sTfR (0.51, *p* < 0.05) and TSAT (0.4, *p* < 0.05).

## Discussion

In the study, we present preliminary data exploring the relationship between iron status and peritoneal membrane transport. We assumed that intraperitoneal milieu and properties of the peritoneal membrane can affect iron homeostasis as a result of local intraperitoneal inflammation and, potentially, clearance of molecules involved in iron metabolism. To our knowledge this is the first study which evaluates iron metabolism biomarkers in dialysis effluent and estimates the relationship between PMT category and biomarkers of iron homeostasis.

Most of the study participants had well controlled anemia. 76% had Hb > 10 g/dL, and the majority of them (69%) had well-balanced iron status. Only 9% patients met the criteria for absolute iron deficiency, while none had functional iron deficiency. Our results stand out significantly from the literature data according to which iron deficiency prevalence exceeds 26% (6, 8, 16). At least in part, this may be due to the fact that current analysis excluded patients with conditions that may have at least potentially contributed to absolute or functional iron deficiency. Fourteen percent of patients in our cohort had high iron status, comparable to other reports (6, 8). These patients tended to have the lowest Hb level despite the highest median CERA dose, and worse preserved RRF. Although the differences were not statistically significant, the characteristics of patients with suboptimal anemia control (Hb < 10 g/dL) and HIS, showed that they tended to have worse preserved RRF and higher levels of ferritin, hepcidin, hsCRP and IL6. The weakness of statistical significance of the observed relationships may result from the relative homogeneity and small size of the group. Nevertheless, strong correlation between GFR and Hb clearly demonstrates the paramount importance of residual renal function in patients with ERSD. It has been ascertained that RRF is associated with higher serum hemoglobin, decreased circulating inflammatory markers, and better clinical outcomes in several regards (17–19).

In the entire group median erythroferrone level was 1.79 ng/mL. Relatively higher levels were observed in patients with high iron status, and ERFE correlated significantly only with hepcidin, but not CERA dose. This finding was unexpected, because physiologically ERFE is produced in response to increased erythropoiesis and increases iron availability via suppression of hepcidin. Nevertheless, ERFE concentration and its significance in CKD patients are not well-characterized, and information on ERFE in CKD is scarce and, in fact, limited to CKD non-dialysis-dependent and hemodialysis patients. In all prior studies positive correlation between serum ERFE levels and serum EPO, as well as ESA dose. Was confirmed, while the relationship between ERFE and biomarkers of iron metabolism has not been clearly demonstrated (20–22). Honda et al. revealed statistically significant negative correlation between ERFE and hepcidin and ferritin levels, and a positive correlation with soluble transferrin receptor (20). The inverse relationship between ERFE and biomarkers of iron metabolism (serum iron and ferritin) was confirmed in the cohort of CKD and HD patients in the Spoto et al. study (22). Meanwhile, in the Honda et al. study, ERFE did not correlate with hepcidin or any biomarker of iron metabolism (21). The modulatory effect of ERFE on iron homeostasis, as well as a role of ERFE as a biomarker of erythropoietic activity in PD patients, and generally in CKD population, need evaluation in future studies.

There is some evidence that systemic and intraperitoneal iron status may differ in patients on peritoneal dialysis (7). However, intraperitoneal iron biology and the impact of individual difference in peritoneal membrane properties on iron status in PD patients are poorly studied.

In our study PMT strongly correlated with dialysate sTfR (0.38, *p* < 0.05), dialysate hepcidin (0.46, *p* < 0.05), dialysate GDF15 (0.5, *p* < 0.01) and dialysate zonulin (0.64, *p* < 0.001) levels, as well as serum IL6 (0.4, *p* = 0.03), serum hs-CRP (0.39, *p* = 0.04) and dialysate hs-CRP (0.38, *p* = 0.04). It is unclear if the abovementioned findings are associated with the properties of the peritoneal membrane and elimination of these molecules via convective transport, or due to systemic and/or local intraperitoneal inflammation. This observation needs to be elucidated further. So far, only peritoneal hepcidin clearance using peritoneal equilibration test was evaluated and confirmed in one small study (15). At the same time, fast peritoneal transport associated with intraperitoneal inflammation results in high peritoneal membrane permeability not only for small solutes but also for middle-size molecules and proteins (23–25).

In our study, fast-average transporters tended to have higher serum and dialysate levels of sTfR, GDF15 and zonulin. Although the differences did not reach statistical significance, fast-average transporters tended to have higher dialysate hepcidin levels, and dialysate hepcidin significantly correlated with serum ferritin (0.38, *p* < 0.05), sTfR (0.51, *p* < 0.05) and TSAT (0.4, *p* < 0.05). Previous studies confirmed the relationship between serum hepcidin and iron status and anemia in patients on PD (14, 26, 27). The significance of this phenomenon is not clear as only one study to date has assessed the concentration of hepcidin in peritoneal effluent (14). In this study, it was found that the removal of hepcidin through the peritoneal membrane is significantly more effective than during hemodialysis or hemodiafiltration, but also that RRF is one of the significant predictors of hepcidin in hemodialysis and peritoneal dialysis patients. Inverse association between hepcidin level and estimated glomerular filtration rate was revealed also in G35–5 CKD patients (28). These findings suggest that hepcidin may be not only key player in iron homeostasis, but also uremic toxin associated with the risk of mortality in PD patients (29).

Soluble transferrin receptor (sTfR) represents the extracellular domain part of the TfR, and its concentration is proportional to the amount of TfR in the total body. STfR is not an acute-phase reactant and is less influenced by inflammation than other iron metabolism indices and may be useful for assessing iron deficiency anemia in chronic disorders. Another known role of sTfR is that it represents the erythropoietic activity of bone marrow (30). In our study population sTfR correlated positively with serum hepcidin and ferritin and dialysate hsCRP and IL6. Our results are in contrary to the study conducted in HD patients (31).

The correlation sTfR with dialysate hsCRP and IL6 may be an expression of specific iron state in peritoneal cavity in PD patients. In Aldriwesh et al. study proteomic analysis revealed that peritoneal transferrin is iron-saturated, which is in marked contrast to transferrin in serum and is a risk of intraperitoneal infection (7).

Zonulin, a 47 kDa protein, which regulates intestinal permeability via alteration of tight junctions, has not been extensively studied in CKD population yielding slightly different results on its function, and in fact there is no data on zonulin in PD patients (32, 33). The study performed in hemodialysis patients revealed significantly increased zonulin levels, as well as inflammatory markers (hsCRP and IL-6) in the study group compared with healthy subjects.

A borderline correlation with serum hypersensitive CRP suggests that zonulin may play a role in the systemic inflammation in HD patients (34). In our study concentration of zonulin in dialysate was significantly higher in fast-average transporters, which corresponded positively with the degree of inflammation as measured by serum hsCRP and IL-6, although with former it did not reach statistical relevance. This finding is of interest, since it reinforces results from the aforementioned study. Further investigation on the role of zonulin in intestinal permeability and induction of inflammation in PD patients would be advisable.

These data could suggest that zonulin could be a link between inflammation and iron homeostasis disorders. However the studies conducted in early stages CKD patients and kidney transplant recipients showed no relationship between zonulin and iron state parameters (35–37).

Growth differentiation factor 15 (GDF15), an anti-inflammatory cytokine, secreted by matured erythroblasts is involved in hepcidin metabolism and as such is potentially involved in iron metabolism. Nevertheless, available data on the role of GDF15 as the marker of iron status in CKD patients are scarce and yield slightly inconsistent results (38–41).

GDF-15 was not studied in adult PD patients, in pediatric PD population it was shown to be elevated in comparison to the healthy subjects and hemodialyzed children (42). The authors did not assess GDF-15 in relation to the modality of PD or membrane transport in PET test.

Our study did not reveal a statistically significant difference between 2 groups of peritoneal membrane transporters. In addition, for the first time we did assess GDF-15 in serum and dialysate in adult PD population.

### Limitations

There are some limitations to our study. This is a single center study. The sample size was relatively small when compared with studies on HD patients, As a result some of our findings did not reach statistical significance. However on the other hand, this is a relative large and homogenous PD population. Despite that, further investigation in larger cohorts from different centers may confirm at least some of our results and may yield others.

## Conclusion

Anemia in PD patients is less pronounced in those with better preserved residual kidney function. This group of patients tends to have a various degree of inflammation, with fast-average transporters displaying greater degree of inflammation. Higher concentration of dialysate sTfR, hepcidin, GDF15 and zonulin in this group of patients may indicate more effective clearance of these molecules. The role of ERFE in regard to erythropoietic activity in PD patients requires further investigation.

## Data availability statement

The original contributions presented in the study are included in the article/supplementary material, further inquiries can be directed to the corresponding author.

## Ethics statement

The studies involving human participants were reviewed and approved by Ethics Committee at Medical University of Warsaw, Poland. The patients/participants provided their written informed consent to participate in this study.

## Author contributions

TG and EW contributed to the conception and design of the study, collected the data, performed the statistical analysis, wrote the manuscript, and prepared its final version. JM contributed to the conception and design of the study, wrote the manuscript, and prepared its final version All authors contributed to the article and approved the submitted version.

## Conflict of interest

The authors declare that the research was conducted in the absence of any commercial or financial relationships that could be construed as a potential conflict of interest.

## Publisher’s note

All claims expressed in this article are solely those of the authors and do not necessarily represent those of their affiliated organizations, or those of the publisher, the editors and the reviewers. Any product that may be evaluated in this article, or claim that may be made by its manufacturer, is not guaranteed or endorsed by the publisher.
